# Evaluation of Pressure Pain Threshold as a Measure of Perceived Stress and High Job Strain

**DOI:** 10.1371/journal.pone.0167257

**Published:** 2017-01-04

**Authors:** Lisbeth Hven, Poul Frost, Jens Peter Ellekilde Bonde

**Affiliations:** 1 Department of Occupational and Environmental Medicine, Bispebjerg Frederiksberg University Hospital, Copenhagen, Denmark; 2 Department of Occupational Medicine, Danish Ramazzini Centre, Aarhus University Hospital, Aarhus, Denmark; University of Würzburg, GERMANY

## Abstract

**Objective:**

To investigate whether pressure pain threshold (PPT), determined by pressure algometry, can be used as an objective measure of perceived stress and job strain.

**Methods:**

We used cross-sectional base line data collected during 1994 to 1995 within the Project on Research and Intervention in Monotonous work (PRIM), which included 3123 employees from a variety of Danish companies. Questionnaire data included 18 items on stress symptoms, 23 items from the Karasek scale on job strain, and information on discomfort in specified anatomical regions was also collected. Clinical examinations included pressure pain algometry measurements of PPT on the trapezius and supraspinatus muscles and the tibia. Associations of stress symptoms and job strain with PPT of each site was analyzed for men and women separately with adjustment for age body mass index, and discomfort in the anatomical region closest to the point of pressure algometry using multivariable linear regression.

**Results:**

We found significant inverse associations between perceived stress and PPT in both genders in models adjusting for age and body mass index: the higher level of perceived stress, the lower the threshold. For job strain, associations were weaker and only present in men. In men all associations were attenuated when adjusting for reported discomfort in regions close to the site of pressure algometry. The distributions of PPT among stressed and non-stressed persons were strongly overlapping.

**Conclusions:**

Despite significant associations between perceived stress and PPT, the discriminative capability of PPT to distinguish individuals with and without stress is low. PPT measured by pressure algometry seems not applicable as a diagnostic tool of a state of mental stress.

## Introduction

Persistent stress and job strain may be related to the development of cardiovascular and mental disease [1–3]. So far, objective measures with capability of distinguishing individuals experiencing persistent stress or high levels of job strain have not been established. Among neuroendocrine hormones, saliva cortisol is the most tested. Studies of its regulation in subjects with persistent stress or job strain have not provided a consistent picture [4, 5]. Moreover, cortisol may be down regulated due to habituation to high stress levels, and it has a large diurnal variation that makes accurate measurements difficult [6–8]. Recent studies have proposed that acute exposure to psychosocial stressors or the perception of being stressed may cause immediate but transient alterations in pain sensitivity determined by pain thresholds of mechanical pressure stimuli [9,10]. Mechanical pressure if one of many various modulators of quantitative sensory testing [11]. These modulators indicate nociceptive sensibility and have also been proposed to detect generalized nociceptive hypersensitivity [11,12]. Previous studies have found pressure pain thresholds (PPT) to have the highest accessibility and thereby the most likely to be implemented in clinical practice [13]. One study on PPT and stress applied pressure algometry to assess pain thresholds among 26 opera singers before, during and after a performance as a measure of acute stress response [9]. Decreased pain threshold was defined by increased pressure pain sensitivity (PPS) in cutaneous poly modal receptors on specific locations on the skin of the sternum. They found an increase in PPS and thereby a decrease in pain threshold during the opera singers performance with normalization immediately after [9]. A later study including 308 Danish office workers reported higher PPS measurements among participants with persistent stress as compared with non-stressed employees [10]. When using a PPS score above a certain high level as cut point for a positive screening test for persistent stress, however, a modest trade-off between sensitivity and specificity was found. For instance, if a sensitivity of 0.8 was set as a goal, the specificity was only 0.3 [10]. We assume that self-reports of perceived stress at the group level is correlated with the condition that Hans Selye in 1936 defined as “the non-specific response of the body to any demand made upon it” [14]. We therefore find it meaningful to use self-reports *at the group level* as golden standard for alternative measures of persistent stress when the individual reporting of being stressed is ill defined and highly subjective.

In this paper, we examine the usefulness of pressure pain threshold (PPT) measurements as a diagnostic measure of persistent stress as defined by self-reports of perceived stress and job strain, respectively.

## Methods

### Population

We used cross-sectional baseline data collected within the Project on Research and Intervention in Monotonous work (PRIM). During 1994–1995, 4162 workers from workplaces throughout Denmark were approached by 3 regional hospital departments of occupational medicine. The workplaces included 4 food processing companies, 3 textile plants, 4 electronic plants, 3 cardboard industries, 1 postal sorting center, 1 bank and 2 supermarkets [15]. All employed female and male workers were eligible for participation, and 3123 of the 4162 workers (75%) completed the baseline questionnaire [16]. The relevant Danish regional Ethical Committees in the counties of Copenhagen, Aarhus, Viborg, Ringkjøbing, Sønderjylland and Fyn approved the PRIM project before its baseline execution in 1994 and all participants provided informed written consent.

### Perceived stress and job strain

To determine perceived stress, we applied questions from by The Stress Profile [17]. The Stress Profile contains 194 questions in total of which 18 concern stress symptoms. The questions comprise somatic symptoms (6 questions), emotional symptoms (8 questions) and cognitive symptoms (4 questions). We used all 18 questions and asked the participants to answer them in consideration to the past 4 weeks and hereby we include all original items of The Stress Profile that address stress symptoms. Each item was then rated on a five point scale ranging from ‘‘very often” (1) to ‘‘never” (5). All items were dichotomized such that item response values 1–2 were assigned a value of 1, whereas response values 3–5 were assigned a value of 0. Subsequently the dichotomized values were summed within each of the three dimensions and standardized to a 4 point scale giving a total scoring across the three dimensions ranging from 0 to 12. Stress scores of ′0′ were observed in 50% of the population and were labelled ‘low’, scores >0–2.66 were observed in 40% and were labelled ‘minor’ and scores above 2.66, observed in 10% of the population, were labelled ‘high’ stress.

Information on job strain was obtained by questions taken from The Whitehall II version of the Job Content Questionnaire [18]. The questions focused on job control (14 questions) and psychological demands (3 questions). The answers were dichotomized into raw scores of either 0 or 1 with 0 being responses ‘never’ or ‘sometimes’ and 1 being the response ‘often’ or ‘very often’. High demands were defined by a sum score across the three items of at least 2 and low control by a sum score across 14 items of 9 or less. If the participant complained of both high demand and low control, psychological strain was defined as being present.

### Discomfort

Preceding the pressure algometry measurements, we obtained information about discomfort in specific anatomical regions. Participants were told to inform of any discomfort in the past 7 days using a questionnaire with drawings of the body regions. Answers were provided on visual analogue scale spanning 0 (no discomfort) and 9 (worst level of discomfort). Due to our specific research concerns, we included information on discomfort in the neck, right and left shoulder and lower back.

### Pressure algometry

Pressure algometry measurements were performed to assess PPTs on the trapezius and supraspinatus muscles and the tibia. Measurements of trapezius and supraspinatus muscles were performed bilaterally and the median values were applied in the analyses. Measurement of PPT of the tibia was performed only on the right tibia in 1074 of the 3123 subjects. PPT (in kPa) was defined as the pressure where pain was first experienced. PPT was measured with an algometer (Somedic, Stockholm, Sweden). Pressure was applied manually by the researchers with an increase-rate of 50 kPa/s through a circular rubber coated pressure probe (1 cm^2^). When subjects reached their PPT they were instructed to push a button which automatically locks the algometer display at the exact value of PPT [19].

### Statistical methods

Using multiple linear regression stratified by gender, we analyzed PPT for each site as a function of persistent stress and job strain, respectively. Persistent stress was analyzed as a categorized variable divided in to three groups using the category as the reference. The performance of the 18 questions on the Setterlind stress scale was evaluated by computing Cronbach-alfa values, and job strain was dichotomized as ‘yes’ (high demands and low control, cf. above) or ‘no’. Results were expressed as crude and adjusted mean values by exposure group and as differences in means across exposure groups with 95% confidence intervals. Adjustments were made for a fixed set of a priory selected extraneous covariates including age, education, smoking status, BMI, and discomfort in an adjacent anatomical region. Discomfort was included as a continuous variable taking the values from 0 to 9. We used discomfort in the anatomical region most closely to the actual site of the PPT (neck for the trapezius region, average of right and left shoulder region for supraspinatus, and low back for tibia). In additional analyses, discomfort in regions more distant to the site of PPT was included (for instance substituting discomfort of the shoulder with discomfort in the low back in models addressing PPT of supraspinatus). All analyses were performed using SAS software Version 9.4 (SAS Institute Inc., Cary, NC, USA) using the GLM (General Linear Model) procedure, which accommodates models with categorical independent variables. Model fits were evaluated by inspection of scatter plots of residual values on the vertical axis and predicted values on the horizontal axis. Although PPT values were not perfectly normally distributed, scatter plots of the residuals were symmetrical around zero indicating acceptable model fits.

## Results

The study included data from 3123 participants (42% women, 58% men), who participated in a questionnaire concerning perceived stress as well as job strain. PPTs were obtained for 1256 men and 1773 women as part of the clinical examination protocol. Table 1 gives the characteristics of the study population according to perceived stress and reporting of job strain, respectively.

**Table 1:** Percentage distribution of study population characteristics according to exposure category.

	Perceived stress, scale 0–12			Job strain	
	0 (n = 1594)	>0–2.66 (n = 1099)	2.67–12.00 (n = 292)	No (n = 2817)	Yes (n = 249)
Men, %	46.2	37.4	35.3	41.8	34.9
Women %	53.8	62.6	64.7	58.2	65.1
Age, years, %					
< 30	25.4	23.9	14.7	24.5	14.9
30–40	28.6	28.0	29.5	28.8	24.5
40+	46.0	48.0	55.5	46.7	60.2
Body mass index, kg/m^2^, %					
< 25	60.0	56.4	61.0	59.1	54.2
25–30	27.3	29.3	24.0	27.7	29.3
30+	8.7	11.7	9.3	9.4	12.1
Education, %					
Skilled worker	30.7	27.8	24.3	28.7	29.7
Unskilled worker	52.6	56.1	57.9	54.5	56.2
Other	13.1	13.7	14.7	13.4	10.8
Smoking, %					
Never	31.8	25.9	22.3	28.3	32.1
Previously	17.8	18.5	19.5	17.9	22.5
Current	50.2	55.4	58.2	53.4	45.0

Tables 2 and 3 show the associations between perceived stress and regional PPTs for men and women, respectively. For all PPT sites in men, adjusted and unadjusted analyses showed significant dose-response relationship between perceived stress and PPT; the higher the stress score, the lower the PPT. Associations were strongly attenuated when experience of discomfort in the region close to the PPT site in addition to age and body mass index were included into the models. Discomfort in regions more distant from the PPT site (for instance, substituting discomfort of the shoulder with discomfort in the low back in models addressing PPT in the supraspinatus) produced almost the same attenuation (results not shown). For all locations, men on average had higher PPT compared with women. In women we found significant results when adjusting for age and body mass index, but not when including discomfort. In both men and women we found the distributions of PPT among stressed and non-stressed persons are strongly overlapping.

**Table 2:** Crude and adjusted means and mean differences of pressure pain thresholds assessed by pressure algometry in the trapezius and supraspinatus muscles and the tibia according to perceived stress score. Men.

		Pressure pain threshold (kPa)							
	Perceived stress score_a_range 0–12	N	Mean_Cr_	SD	Mean_Adj1_	ΔMean_Adj1_	Mean_Adj2_	ΔMean_Adj2_	95% CI
M. Trapezius	0 (reference)	709	590.6	253.5	564.6	-	556.5	-	-
	>0–2.66	400	541.9	238.4	544.8*	19.8*	550.6	5.8	-23.8–35.5
	2.67–12.00	98	491.5	216.7	469.8*	94.8*	499.0*	57.5*	4.3–110.6
M. Supraspinatus	0 (reference)	707	699.5	261.8	704.8	-	698.2	-	-
	>0–2.66	399	676.4	251.1	679.5*	25.3*	684.1	14.2	-15.6–44.6
	2.67–12.00	98	626.9	248.6	602.3*	102.5*	641.0*	57.2*	2.2–112.2
Tibia	0 (reference)	705	732.3	239.6	733.1	-	729.7	-	-
	>0–2.66	399	709.9	233.8	710.8*	22.3*	714.0	15.7	- 14.1–45.5
	2.67–12.00	97	660.7	204.8	642.9*	90.2*	660.7*	69.0*	15.1–122.9

*: P < 0.05

MeanAdj1: Adjusting for age and body mass index.

MeanAdj2: Adjusting for age, BMI body mass index and discomfort from the region closest to the anatomical site of pressure algometry

ΔMean: Comparing middle vs. lowest and highest vs. lowest stress scores, respectively.

**Table 3:** Crude and adjusted means and mean differences of pressure pain thresholds assessed by pressure algometry in the trapezius and supraspinatus muscles and the tibia according to perceived stress score. Women.

		Pressure pain threshold (kPa)							
	Perceived stress score_a_range 0–12	N	Mean_Cr_	SD	Mean_Adj1_	ΔMean_Adj1_	Mean_Adj2_	ΔMean_Adj2_	95% CI
M. Trapezius	0 (reference)	838	344.3	161.9	343.0	-	338.1	-	-
	>0–2.66	667	327.5	146.3	327.2*	15.8*	330.4	7.7	-8.2–23.6
	2.67–12.00	180	315.2	135.9	316.1*	26.9*	331.4	6.6	-19.1–32.4
M. Supraspinatus	0 (reference)	838	438.1	174.9	463.3		431.0		
	>0–2.66	333	421.1	167.6	421.6*	14.7*	423.1	7.9	-9.7–25.4
	2.67–12.00	180	413.7	163.3	413.2*	23.1*	431.4	-0.4	-29.1–28.3
Tibia	0 (reference)	830	497.7	172.7	494.4		490.0		
	>0–2.66	662	469.6	176.5	471.4*	23.0*	471.3*	18.7	0.6–36.7
	2.67–12.00	178	458.6	156.8	457.5*	37.0*	472.1*	17.9	-11.5–47.2

*: P < 0.05

MeanAdj1: Adjusting for age and body mass index.

MeanAdj2: Adjusting for age, BMI body mass index and discomfort from the region closest to the anatomical site of pressure algometry

ΔMean: Comparing middle vs. lowest and highest vs. lowest stress scores, respectively.

The associations between job strain and PPT are depicted in Tables 4 and 5 for men and women. In men the differences were significant for all PPT sites except the tibia. The associations between job strain and PPT in women showed no specific pattern and none of the differences reached statistically significance.

**Table 4:** Crude and adjusted means and mean differences of pressure pain thresholds assessed by pressure algometry in the trapezius and supraspinatus muscles and the tibia according to self-reported job strain. Men.

		Pressure pain threshold (kPa)							
	Self-reported job strain	N	Mean_Cr_	SD	Mean_Adj1_	ΔMean_Adj1_	Mean_Adj2_	ΔMean_Adj2_	95% CI
M. Trapezius	No (reference)	1136	553.2	246.5	555.2		553.6		
	Yes	85	480.4	228.1	477.8*	77.4*	490.5	63.0*	10.3–115.8
M. Supraspinatus	No (reference)	1133	691.5	256.7	694.4		693.9		
	Yes	85	601.4	245.2	596.9*	97.5*	617.7	76.1*	22.3–130.0
Tibia	No (reference)	1130	722.2	235.5	721.9		721.8		
	Yes	85	663.6	226.5	660.4*	61.5*	669.3	52.4	-0.5–105.4

*: P < 0.05

MeanAdj1: Adjusting for age and body mass index.

MeanAdj2: Adjusting for age, BMI body mass index and discomfort from the region closest to the anatomical site of pressure algometry

ΔMean: Comparing high job strain with low job strain

**Table 5:** Crude and adjusted means and mean differences of pressure pain thresholds assessed by pressure algometry in the trapezius and supraspinatus muscles and the tibia according to self-reported job strain. Women.

		Pressure pain threshold (kPa)							
	Self-reportedjob strain	N	Mean_Cr_	SD	Mean_Adj1_	ΔMean_Adj1_	Mean_Adj2_	ΔMean_Adj2_	95% CI
M. Trapezius	No (reference)	1595	331.8	144.9	331.2		330.4		
	Yes	156	356.9	212.3	351.4	-20.2	354.5	-23.6	-49.2–2.0
M. Supraspinatus	No (reference)	1594	426.8	171.6	425.8		425.2		
	Yes	156	442.1	157.4	437.9	-12.1	442.6	-17.4	-45.9–11.1
Tibia	No (reference)	1582	484.7	175.4	482.3		481.0		
	Yes	153	461.4	150.4	471.6*	9.7*	477.0	2.0	-27.6–31.6.

*: P < 0.05

MeanAdj1: Adjusting for age and body mass index.

MeanAdj2: Adjusting for age, BMI body mass index and discomfort from the region closest to the anatomical site of pressure algometry

ΔMean: Comparing high job strain with low job strain

## Discussion

This study finds perceived stress and job strain to be associated with lower PPT values in the trapezius, the supraspinatus as well as the tibia in men as well as women. In men differences in PPT were markedly reduced after inclusion of self-reported discomfort, which indicates a correlation between regional pain and lower PPT.

In considering the relation between job strain and PPT, this study found the tibia to be the only site not to reveal significant associations. Nociceptors in periosteum have shown to respond well to pressure stimulation, however muscle stimulation have been suggested more likely to succumb to hypersensitization [20]. Hypersensitization plays a part in many chronic pain disorders such as fibromyalgia syndrome (FMS), which has been associated with substantial decrease in pain threshold [21, 22]. One limitation in this study is that the exclusion criteria in the PRIM study did not include these pain disorders, however when including reports of discomfort the subjects with FMS would most likely be without substantial impact on the reported associations.

According to earlier studies, PPT may be altered in other chronic diseases as well. Myocardial infarction, COPD and diabetes might result in biased associations due to confounding [23]. However, since the study population only includes individuals that gainfully employed at the time of the examination the prevalence of generalized pain disorders as myalgia and other disabling chronic disease is very low and thus confounding by comorbidity is less likely.

The studies by Ballegaard et al. have also investigated whether transient and/or persistent stress show alterations in pain sensitivity. They suggest that pressure algometry can be used as an objective marker of transient stress when applying pressure on identified areas on the skin over the sternum with certain pain receptors. The authors hypothesize these pain receptors to have specific relation to the heart and, consequently, the ability to become sensitized in both transient and persistent stress [9, 10, 24]. Our study did not include pressure algometry on the skin of the sternum. Furthermore, the pressure algometry applied in the PRIM Study was performed with attempt to stimulate pain receptors in the muscle tissue and not the skin. Spatial summation, an increase in pain perception when larger areas of stimulation are used is considered an important factor in terms of pain threshold measurements [25]. In the PRIM Study, the pressure was conducted through a probe of 1cm^2^. As this is considered a small area it is not likely to succumb to spatial summation. The studies by Ballegaard et al. did not mention data on probe size.

In the PRIM Study PPT was defined as the point where participants began to sense pain from the pressure. This is in accordance with previous reported observations of positive correlation between PPS and higher symptoms score [10]. Furthermore, this study has been conducted on an all working population and with overall low stress scores. To test, and possibly strengthen, our findings we suggest doing a study with participants, who have a reported high level of stress, to such a degree that the condition is withholding them from maintaining employment. We also advise that the term ‘objective’ should be applied with caution. Although PPT was measured by an objective device (the pressure algometer), the participants themselves were asked to signal the point which the pressure activated their pain receptors.

Among the main advantages of this study is that it has been conducted on a large population. It also presents several significant findings when stratifying for gender and adjusting for the highly relevant confounders of pain threshold, age, BMI as well as reported discomfort [23, 26]. However we find that the distributions of pain thresholds among stressed and non-stressed persons are strongly overlapping, causing a discriminative value too low to allow for a distinguishing of stressed from non-stressed participants.

## Conclusions

Despite significant associations between perceived stress and PPT in both men and women, we find a discriminative value too low to suggest pain thresholds measured by pressure algometry as a diagnostic tool of stress.

## Supporting Information

S1 Dataset Anonymized original individual level data from the PRIM cohort. Data includes a subset of 15 variables used for the analyses presented in this paper.
(SAS7BDAT)
